# Development of Thyme-Infused Polydimethylsiloxane Composites for Enhanced Antibacterial Wound Dressings

**DOI:** 10.3390/ma17174224

**Published:** 2024-08-27

**Authors:** Sara Sarraj, Małgorzata Szymiczek, Anna Mertas, Agata Soluch, Dariusz Jędrejek, Sebastian Jurczyk

**Affiliations:** 1 Department of Theoretical and Applied Mechanics, Silesian University of Technology, Konarskiego 18A Str., 44-100 Gliwice, Poland; malgorzata.szymiczek@polsl.pl; 2 Department of Microbiology and Immunology, Faculty of Medical Sciences in Zabrze, Medical University of Silesia in Katowice, Jordana 19 Str., 41-808 Zabrze, Poland; amertas@sum.edu.pl; 3 Department of Biochemistry and Crop Quality, Institute of Soil Science and Plant Cultivation—State Research Institute, Czartoryskich 8 Str., 24-100 Puławy, Poland; asoluch@iung.pulawy.pl (A.S.); djedrejek@iung.pulawy.pl (D.J.); 4 Łukasieiwcz Research Network—Institute for Engineering of Polymer Materials and Dyes, M. Sklodowska-Curie 55 Str., 87-100 Toruń, Poland; sebastian.jurczyk@impib.lukasiewicz.gov.pl

**Keywords:** polydimethylsiloxane, wound dressings, organic filler, thyme, antibacterial activity

## Abstract

Polydimethylsiloxane (PDMS) is widely used in biomedical applications due to its biocompatibility and flexibility but faces challenges due to its hydrophobicity and limited mechanical strength. This study explores the incorporation of thyme (*Thymus vulgaris* L.) into PDMS to enhance its properties for wound dressing applications. PDMS composites containing 2.5 wt.% and 5 wt.% of thyme were prepared and evaluated for physical, chemical, mechanical, and biological properties. Scanning electron microscopy, contact angle measurements, absorption tests, Fourier-transform infrared spectroscopy, differential scanning calorimetry, hardness, tensile testing, antibacterial activity, and cell viability assays were conducted. Thyme integration improved mechanical properties with increased absorption and preserved hydrophobicity. FTIR and DSC analyses indicated minimally altered crystallinity and chemical interactions. Hardness decreased with higher thyme content due to terpene-induced polymerization inhibition. Tensile testing showed reduced stress at break but increased elongation, suitable for wound dressings. Enhanced antibacterial activity was observed, with composites meeting bacteriostatic standards. Cell viability exceeded 70%, with optimal results at 2.5 wt.% thyme, attributed to cytokine-inducing compounds. Thyme-incorporated PDMS composites exhibit improved antibacterial and mechanical properties, demonstrating the potential for advanced wound dressings.

## 1. Introduction

Recent advancements in cellular biology have substantially deepened our comprehension of wound healing, resulting in enhanced wound care methodologies. The typical response to injury is characterized by four overlapping phases: hemostasis, inflammation, proliferation, and remodeling, which are observed across all organ systems [1]. During the inflammatory phase, hemostasis is established, further blood loss is prevented, and inflammatory cells migrate to the injury site. This is followed by the proliferative phase, which includes wound granulation, fibrogenesis, wound contraction, neovascularization, and re-epithelialization [2]. Wounds, defined as disruptions of the epidermal continuity, can be categorized as acute or chronic depending on the duration of the healing process. If not properly managed, both types can pose significant health risks, with skin and soft tissue infections being a primary cause of delayed healing [3,4]. An important aspect of wound care is their ability to promote cell proliferation, fight the surrounding microbes, and stop them from forming biofilm. One of the most popular group of materials used in this field is organosilicon polymers.

Polydimethylsiloxane (PDMS) is a versatile silicon-based polymer extensively utilized in biomedical applications due to its excellent biocompatibility, flexibility, and gas permeability [5]. These attributes make PDMS an attractive candidate for wound dressing materials. However, its inherent hydrophobicity and limited mechanical strength pose significant challenges to its direct application in wound care. The hydrophobic nature of PDMS can hinder cell adhesion and proliferation, which are critical for effective wound healing. At the same time, its insufficient mechanical robustness can limit its durability and performance in dynamic biological environments [6,7].

To overcome these limitations, researchers have explored the incorporation of various fillers into the PDMS matrix [8,9]. By integrating fillers, the modified PDMS composites aim to combine the advantageous properties of both PDMS and the fillers, resulting in improved wound dressing materials.

Nevertheless, the scientific community is increasingly focusing on using organic fillers rather than inorganic ones. These fillers, derived from natural sources, are recognized for their biocompatibility, biodegradability, and ability to enhance cell adhesion, proliferation, and differentiation. Numerous researchers have successfully incorporated various naturally derived fillers into polymeric matrices to enhance bioactivity while maintaining the necessary operational properties specific to their fields. The prevailing trend centers on integrating extracts, predominantly from plants, into polymeric matrices. For instance, one study of electrospun polydimethylsiloxane (PDMS) with chitosan demonstrated the positive impact of this incorporation on the wound-healing process [10]. Other studies have coated the matrix with a layer of herbal extracts, achieving accelerated wound healing [11]. A similar approach by Ponrasu et al. utilized a mixture of herbal extracts, including green tea extract known for its high anti-inflammatory and antimicrobial properties, to promote re-epithelialization [12]. Sreekantan et al. achieved groundbreaking results by composing a silica coating from palm oil fuel ash that exhibited hydrophobicity while maintaining low cell toxicity, depending on the concentration [13]. In one of our previous works [14], we successfully developed sage–PDMS composites that exhibit antibacterial activity against Staphylococcus aureus, demonstrating that such fillers can benefit both patients and the environment by reducing the harmful production steps compared to inorganic fillers.

The integration of organic fillers into PDMS represents a promising approach to developing advanced wound dressings that address the current limitations of PDMS. These composites have the potential to provide a moist wound environment, promote cell adhesion and proliferation, offer antibacterial properties, and demonstrate environmental friendliness.

Considering this objective, the authors sought to develop herb-based composites utilizing an organosilicon polymer matrix specifically designed to enhance antibacterial properties for wound dressing applications. To achieve this, thyme (*Thymus vulgaris* L.) was selected as the modifying additive due to its well-documented medicinal benefits. Thyme, a herbaceous perennial aromatic plant, is extensively utilized in medicine for its antioxidant, antimicrobial, and anti-inflammatory properties [15]. Previous studies have demonstrated that thyme exhibits significant antibacterial activity, particularly against Gram-positive bacteria [16,17].

The developed materials incorporated varying concentrations of thyme and underwent comprehensive physical, chemical, mechanical, and biological evaluations to assess the impact of thyme integration on these properties. This study underscores the potential of these modified PDMS materials in enhancing wound healing outcomes, suggesting a promising future for such composites in clinical wound care applications.

## 2. Materials and Methods

### 2.1. Materials Preparation

For this study, a two-component, medical-grade polydimethylsiloxane (PDMS), specifically Dragon Skin™ 30 (Smooth-On Inc., Macungie, PA, USA), was selected as the composite matrix. Thyme (*Thymus vulgaris* L.) was utilized as a modifier to enhance the matrix’s antibacterial properties. The dried herb underwent an additional drying process for 1 h at 20 °C. Subsequently, the filler was ground using a four-blade grinder and sieved through 100 µm meshes with a Multiserw sieve shaker (MULTISERW-Morek, Brzeźnica, Poland).

**Figure 1:**
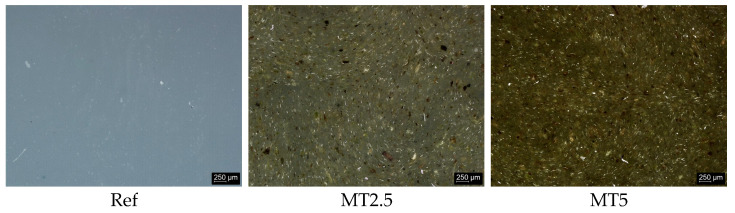
The surface of the acquired materials.

The silicone base was mixed with the prepared filler at 2.5% and 5% weight percentages. Then, the catalyst was added to the mixture in a 1:1 ratio with the base. The resulting mixtures were processed in a rotary mixing machine at 150 rpm for 3 min, followed by vacuum degassing for 4 min to remove any trapped air bubbles and ensure a homogeneous structure. The prepared materials were then gravity cast into molds coated with a thin polyvinyl alcohol layer to facilitate easy demolding. For the purpose of this work, the composite containing 2.5 wt.% filler will be referred to as MT2.5, while the composite containing 5 wt.% filler will be referred to as MT5. Figure 1 presents the surface of the samples taken on a Leica DVM6 stereomicroscope (Leica Microsystems, Wetzlar, Germany).

### 2.2. Researching Methods

The morphological features of the chosen filler were investigated using a Nova Nano SEM 200 scanning electron microscope (FEI, Hillsboro, OR, USA). Before analysis, the filler particles underwent coating with platinum powder for 90 s to improve their conductivity and visibility under the microscope. Observations were made at different magnifications to ensure a thorough examination.

Particle size and grain distribution were evaluated using laser diffraction (LD) with a Fritsch Analysette 22 Micro Tec Plus system (FRITSCH GmbH, Idar-Oberstein, Germany).

The total phenolic content (TPC) of thyme aqueous extract was measured using the Folin–Ciocalteu assay. In this method, 100 µL of Folin–Ciocalteu reagent was added to 1.6 mL of a diluted sample (0.2–1.0 µg/mL) or a gallic acid standard solution (1.0–10 µg/mL). Then, 0.3 mL of Na_2_CO_3_ (15% *w*/*v*) was added, and the mixture was incubated at 40 °C for 30 min. Absorbance was measured at 765 nm using an Evolution 260 Bio spectrophotometer (Thermo Fisher Scientific, Waltham, MA, USA). The TPC was calculated from the linear calibration curve of gallic acid (R^2^ > 0.998) and expressed as milligrams of gallic acid equivalents per gram of dry weight (mg GAE/g DW).

The identification and quantification of the phytochemical composition of thyme were conducted using high-performance liquid chromatography coupled with mass detection (HPLC-MS) analysis. This method utilized a Thermo Ultimate 3000RS chromatography system (Thermo Fisher Scientific, Waltham, MA, USA) equipped with a corona-charged aerosol detector and coupled with a Bruker Impact II HD quadrupole-time of flight mass spectrometer (Bruker, Billerica, MA, USA). The test was carried out in accordance with the comprehensive methodological details outlined in a prior study [14].

The surface wettability of the samples was assessed by measuring the water contact angle (WCA) using an Attension Theta Flex tensiometer (Biolin Scientific, Gothenburg, Sweden). These measurements were performed in accordance with the EN 828:2013 standard [18], employing the sessile drop method with distilled water as the medium. Five droplets, each with a volume of 2 µL, were deposited on the surface of each test sample. The duration of each measurement was 60 s, with a sampling frequency of 1 Hz. The tests were conducted on round samples with a diameter of 16 mm and a thickness of 4 mm.

The absorption evaluation was performed on samples immersed in an artificial plasma solution, prepared according to the ISO 10993-15 standard (Table 1) [19], miming human extracellular fluid and approximating operational conditions. The samples were incubated for 7 days at 35 ± 1 °C. Every 24 h, three samples of each material were removed, weighed, and then returned to the solution for continued observation. The test procedure followed the ISO 62 standard [20], and the absorption was calculated based on the following Equation (1):

(1) $$A_{w}=\frac{m_{2}{-m}_{1}}{m_{1}}\times 100$$

where $m_{1}$ (g) is the sample mass before incubation and $m_{2}$ (g) is the sample mass after incubation.

Fourier transform-infrared spectroscopy (FT-IR) spectra were obtained in attenuated total reflection (ATR) mode using the IRSpirit spectrophotometer (Shimadzu Corporation, Kyoto, Japan). The spectra were collected across the mid-infrared range (4000–400 cm^−1^) with a resolution of 4 cm^−1^ and an average of 20 scans. Before each test, a background spectrum against air was recorded. Following each test, the ATR diamond crystal was cleaned with ethanol.

Differential scanning calorimetry (DSC) analysis of the acquired materials was conducted adhering to ISO 11357-1 [21] using a DSC 1 differential scanning calorimeter (Mettler Toledo, Greifensee, Switzerland). Specimens (20–21 mg) were weighed on an XS105DU analytical balance (Mettler Toledo, Greifensee, Switzerland) and sealed in 40 µL aluminum pans. Each sample was heated from −90 °C to 220 °C at 20 °C per minute and cooled at the same rate in a nitrogen atmosphere (60 mL/min).

The hardness of the samples was determined using a Shore type A durometer (Zorn Stendal, Stendal, Germany). This evaluation was carried out following the ISO 7619-1 standard [22] on samples measuring 50 mm × 50 mm × 4 mm. Each type of material underwent five measurements.

To determine the mechanical strength of the materials, a static tensile test was performed using an AGX kN10D testing machine (Shimadzu Corporation, Kyoto, Japan), following the guidelines outlined in the ISO 527-1 standard [23]. The test was conducted at a crosshead speed of 500 mm/min. The samples, conforming to ISO 527-2–type 5-B specifications, had dimensions of 60 mm in length and 4 mm in width along the measuring length.

To assess the antibacterial properties of the developed materials, an examination was conducted following the ISO 22196 standard [24], with adjustments for sample size. Samples with a diameter of 16 mm were tested against the *Staphylococcus aureus* ATCC 6538P strain. The bacterial strain was initially spread onto Mueller-Hinton agar and incubated at 35 ± 1 °C for 24 h. Following sterilization with 70% ethanol, the samples were placed in a multi-well plate, and 1 mL of a bacterial suspension (1.5 × 10^8^ CFU/mL) was applied to each sample. The specimens were then incubated at 35 ± 1 °C for another 24 h. After incubation, the samples were washed with 1 mL of SCDLP broth and serially diluted. Each dilution and the control were spread onto agar plates and incubated at 35 ± 1 °C for 24 h. Colony counts were then performed. Bacterial reduction was calculated using the following Equation (2):

(2) $$R=\left(U_{t}-U_{0}\right)\left(A_{t}-U_{0}\right)=U_{t}-A_{t}$$

where $U_{0}$ (cells/cm^2^) is the initial viable bacteria log, $U_{t}$ (cells/cm^2^) is the viable bacteria log from the untreated sample after 24 h of inoculation, and $A_{t}$ (cells/cm^2^) is the viable bacteria log from the treated sample after 24 h of inoculation.

The cell viability assay followed ISO 10993-5 standards [25]. Extracts from the tested materials were prepared by placing each sample in a plate with 2 mL of the L-929 fibroblast culture medium and incubating at 37 ± 1 °C with 5% CO_2_ for 2 and 10 days. Control media without samples were similarly incubated. Both extracts and controls were stored at −80 °C until use.

Mouse fibroblasts from the L-929 line (NCTC clone 929) were cultured in Eagle’s Minimum Essential Medium (EMEM) with 10% horse serum, penicillin (100 IU/mL), and streptomycin (100 µg/mL) in 25 cm^2^ polystyrene flasks at 37 °C, 5% CO_2_, and 100% humidity. Cells were passaged every 2–3 days, and a suspension of 1 × 10⁵ cells/mL was prepared for testing.

For the cytotoxicity assessment, L-929 fibroblasts were exposed to undiluted composite extracts for 24 h. The MTT assay was used to evaluate cell viability by measuring mitochondrial dehydrogenase activity. A viability reduction of over 30% compared to control cultures (viability under 70%) indicated cytotoxicity.

In the assay, each well received 100 µL of L-929 cell suspension (10,000 cells/well) in RPMI 1640 medium with 10% FBS, penicillin (100 IU/mL), and streptomycin (100 µg/mL). After 24 h of incubation, the supernatants were replaced with 100 µL of either test extract or control medium. Following another 24 h incubation, wells received 1.1 mM MTT solution. After 3 h, supernatants were removed, 200 µL of DMSO was added to dissolve the MTT formazan, and absorbance at 550 nm was measured using an Eon plate reader. The violet color intensity was proportional to the number of viable cells, and cell viability was calculated using the following Equation (3):

(3) $$Cell\;viability\;(\%)=\frac{Sample\;absorbance}{Control\;abrosbance}\times 100$$

All tests were conducted in a controlled environment, maintaining a temperature of 21 ± 1 °C and a humidity level of 40% unless otherwise specified. Data curation and statistical analysis were performed using OriginLab software 2022b (OriginLab Corporation, Northampton, MA, USA).

## 3. Results and Discussion

### 3.1. Filler’s Characterization

**Figure 2:**
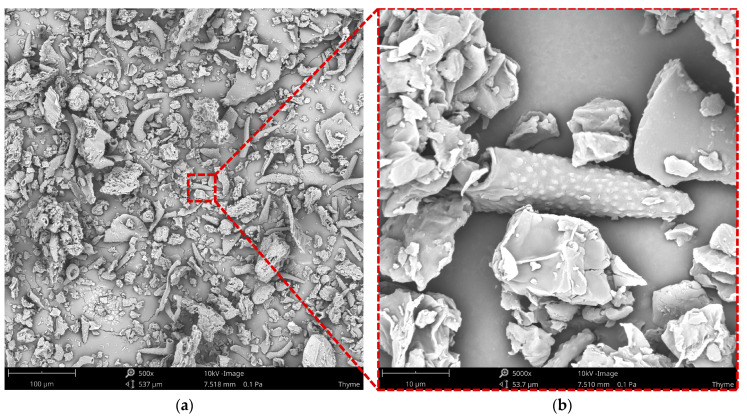
The morphology of thyme at a magnification of 500× (**a**) and 5000× (**b**).

The morphology analysis of thyme, depicted in Figure 2, reveals a diverse array of irregularly shaped particles. The micrographs exhibit plant-specific structures, such as stomata and trichomes. The trichomes are notably short, with a jagged surface attributed to glandular presence. The filler’s highly developed surface area is likely to enhance integration with the matrix, thereby contributing to high mechanical properties.

**Figure 3:**
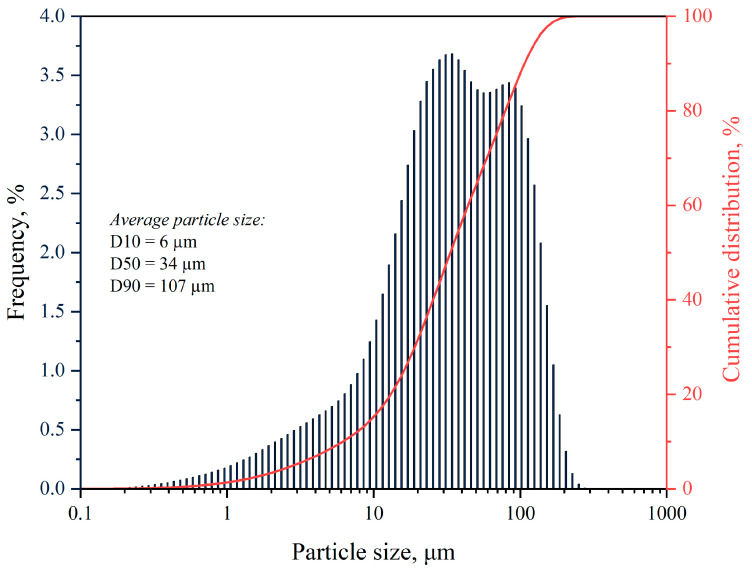
The particle size and distribution results.

The particle size and distribution results, as shown in Figure 3, indicate that despite the filler being sieved through 100 µm meshes, trace amounts of larger particles were still present. Furthermore, over 90% of the grains were smaller than 107 µm. The size and morphology of the filler play a crucial role in determining the properties of the composites.

**Figure 4:**
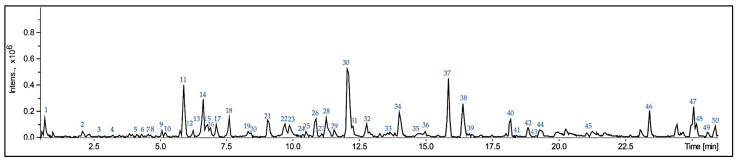
HPLC-MS results.

As depicted in Figure 4, the phytochemical analysis of thyme enabled the identification and quantification of the chemical compounds present in the herb (for a detailed description of the metabolites, corresponding to the presented in Figure 4 numbers, see Table S1. UHPLC-QTOF-MS/MS data of compounds detected in thyme, Supplementary Materials). The total phenolic content (TPC) of thyme was 74.24 ± 2.95 mg GAE/g DW, based on dry weight, which is consistent with findings from other studies [16,26]. Moreover, the phytochemical profile of the herb was dominated by phenolic compounds (e.g., gallocatechin, rosmarinic acid, and salvianolic acid) and terpenes (e.g., carnosol and oxooctadecadienoic acid). The majority of metabolites identified in the samples were compounds previously confirmed to be present in the genus *Thymus*.

### 3.2. Contact Angle Measurements

**Figure 5:**
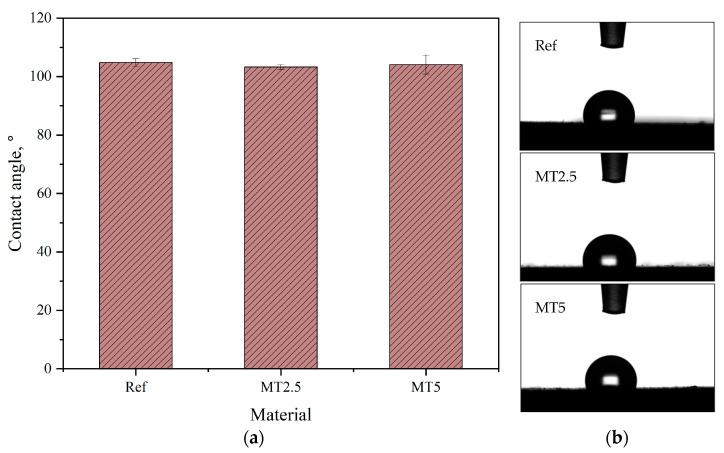
Results of contact angle measurements (**a**) and images of the sessile drops post-test (**b**).

The wettability of the materials was assessed by measuring the water contact angle, as depicted in Figure 5a. Figure 5b presents the sessile drops following the completion of the test. PDMS, known for its hydrophobic surface, retained its hydrophobic characteristics even after the addition of thyme. This is crucial from a medical application perspective, as hydrophobic surfaces are less conducive to microbial attachment and biofilm formation. Similar results were reported by Vagos et al. [27], who demonstrated the positive impact of hydrophobicity on the attachment of bacterial cells. Furthermore, a higher standard error was observed for samples with a greater filler content. This variability may be attributed to the increased likelihood of encountering filler particles on the surface during testing, leading to a broader distribution of contact angle values. However, a one-way ANOVA indicated that the incorporation of thyme into PDMS did not significantly affect the contact angle values (*p* > 0.05).

### 3.3. In Vitro Absorption

**Figure 6:**
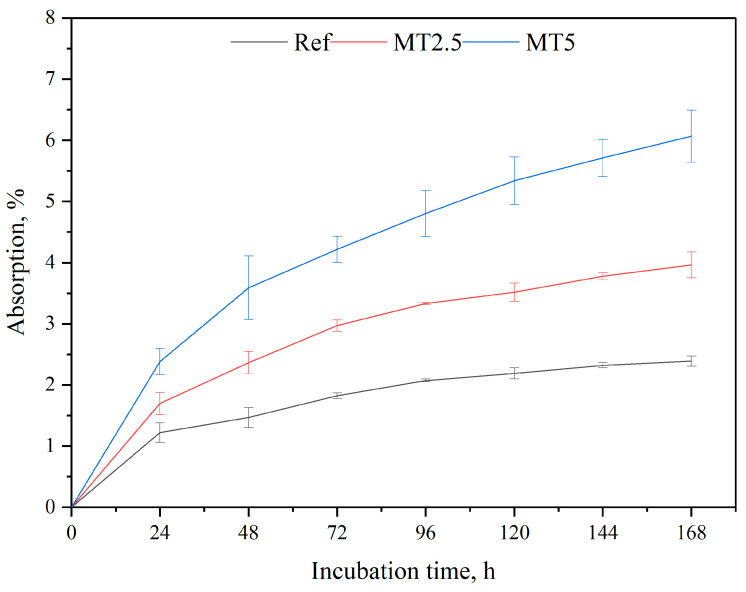
In vitro absorption results.

The absorption characteristics of the tested materials, as shown in Figure 6, indicate that the highest absorption rate occurs within the first 24 h of incubation. The reference samples exhibit an initial steep rise, approaching a plateau but still showing a slight upward trend. In contrast, this phenomenon is not observed in the composites, which demonstrate an increase in absorption at each 24-h interval. This difference is attributed to the highly hygroscopic nature of the filler, resulting in greater absorption of the artificial plasma solution. Additionally, higher filler content correlates with greater variability in the absorption results. The one-way ANOVA analysis proved the significance of the tested property (*p* < 0.05).

### 3.4. Fourier Transform Infrared Spectroscopy (FTIR)

**Figure 7:**
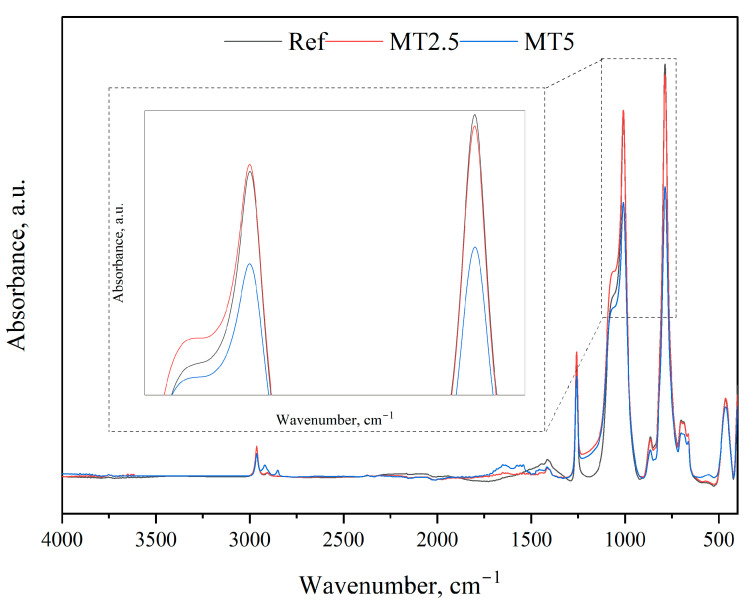
FT-IR spectra of the tested materials.

The IR spectra of the reference material and the composites developed are depicted in Figure 7. It is evident that the addition of the herb did not alter the fundamental chemical structure of PDMS. However, it did affect the intensity of specific peaks: at 779 cm^−1^ and 1251 cm^−1^ which correspond to the CH_3_ rocking and bending in Si-CH_3_, respectively, and at 1008 cm^−1^ attributed to the Si-O-Si band. A notable decrease in intensity was observed in the CH_3_ rocking band, which became more pronounced with an increased content of filler. For the Si-O-Si band, a slight increase in intensity was noted for the composite designated MT2.5, followed by a decrease for MT5. A similar pattern was observed for the CH3 bending in Si-CH3. These spectral changes may have implications for the mechanical performance of the composites.

### 3.5. Differential Scanning Calorimetry (DSC)

**Figure 8:**
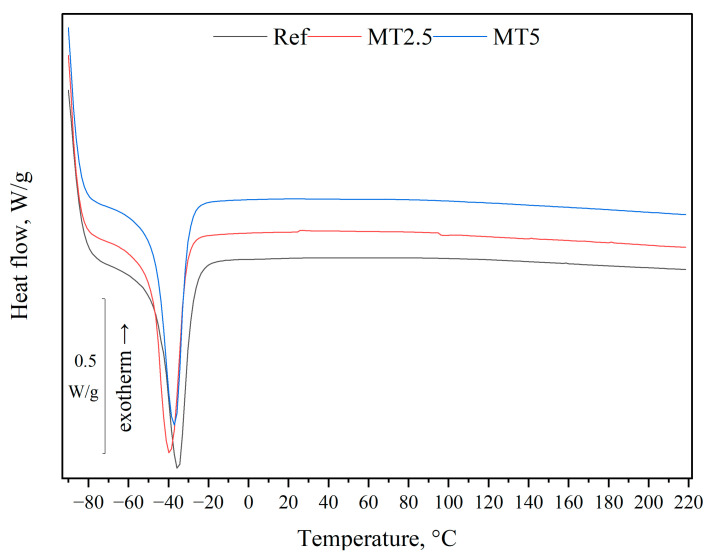
DSC thermograms of the tested materials.

As shown in Figure 8, the incorporation of thyme did not significantly alter the thermal behavior of PDMS, including the melting temperatures. Additionally, no new endothermic or exothermic peaks were observed. However, the addition of thyme did influence the degree of crystallinity, as detailed in Table 2. The crystallinity increased by incorporating 2.5 wt.% thyme but slightly decreased upon adding 5 wt.% of the same filler. This suggests that the chemical compounds present in thyme, illustrated in Figure 4, may have integrated into the chemical structure of the matrix, potentially facilitating an increased nucleation process in the polysiloxane. However, this enhancement depends on the filler content, with the effect reversing when the amount surpasses a certain threshold.

### 3.6. Hardness Measurements

**Figure 9:**
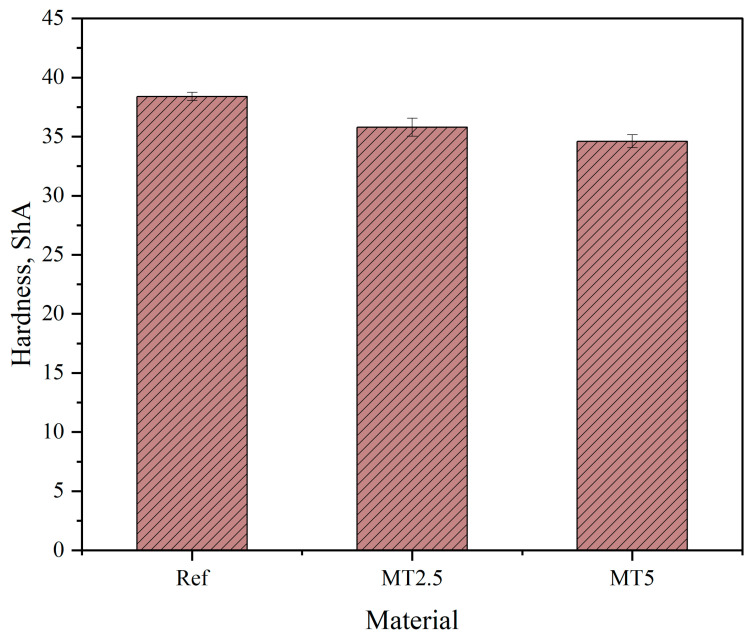
Hardness measurements results.

Figure 9 presents the results of the hardness measurements for the tested materials. Notably, the hardness of the matrix exceeded the manufacturer’s stated value by approximately 28%, which may be attributed to the conditioning process following the gravity casting of the materials. Furthermore, the addition of fillers resulted in a decrease in hardness by 7% for MT2.5 and 10% for MT5. This decrease is likely due to the chemical composition of the filler, which contains a significant number of terpenes that inhibit the polymerization of the matrix, thereby affecting its mechanical properties. Additionally, statistical analysis confirmed that the hardness values were significantly different across samples (*p* < 0.05). However, no significant differences were observed between the composites, suggesting that the incorporation of thyme affects the hardness of the matrix similarly regardless of the filler content.

### 3.7. Tensile Testing

**Figure 10:**
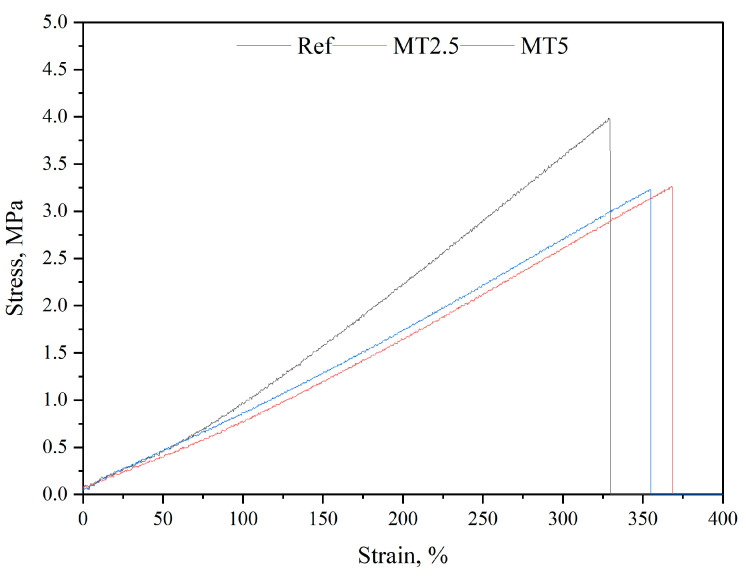
Stress–strain plots of the tested materials.

**Figure 11:**
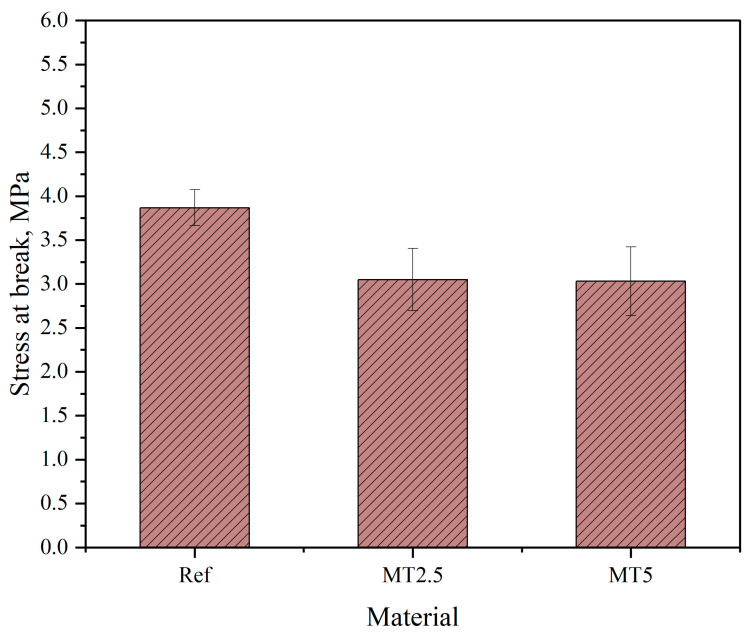
Stress at break results.

**Figure 12:**
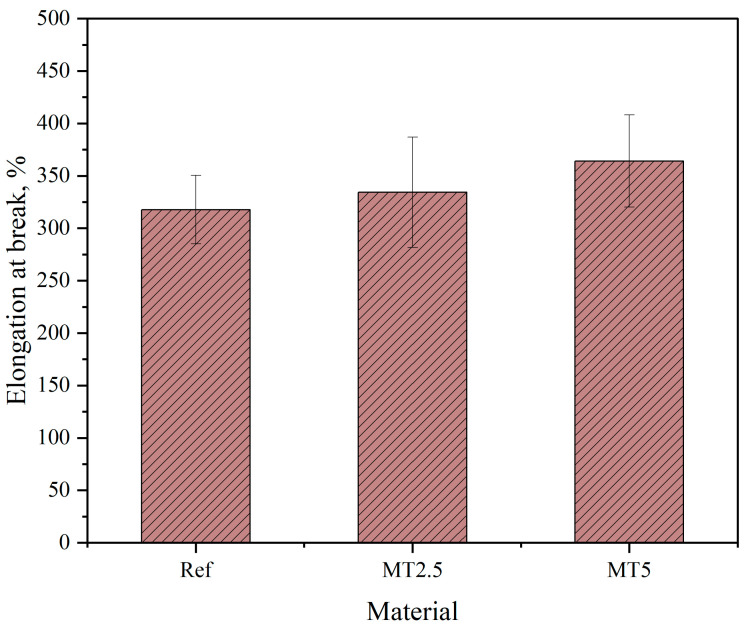
Elongation at break results.

Figure 10 presents stress–strain plots of the obtained materials and Figure 11 and Figure 12 present the results of stress at break and elongation at break, respectively.

The data presented in Figure 11 indicate that the incorporation of thyme into polydimethylsiloxane affects its mechanical strength. Similar to the hardness results, the reduction in stress at break can be attributed to the decrease in the characteristic peaks of PDMS, which is likely due to the high terpene content in thyme. Additionally, the wide variability in the results may be due to the detachment of filler particles during testing, which influences the test outcomes. Furthermore, statistical analysis confirmed the significant impact of the addition of thyme on the stress at break value (*p* < 0.05); however, the composites did not exhibit statistically significant differences. Nevertheless, the stress at break values fell within the acceptable range required for materials used in the production of wound dressings, as reported in other studies [28,29].

As illustrated in Figure 12, the elongation at break increases with the addition of thyme, and this increase is further amplified with higher filler content in the composites. This phenomenon may be attributed to the uniform distribution of the filler within the matrix and the alignment of the longer filler particles along the tensile direction. Additionally, all materials displayed a wide range of error, likely due to the concave formation on the measuring length of the samples caused by punch-cutting, which is a characteristic of the matrix. This concavity may have influenced the materials’ behavior during tensile testing. The one-way ANOVA analysis indicated that these differences were not statistically significant (*p* > 0.05). It is important to note that this range of elongation supports the operational properties required for producing wound dressings.

### 3.8. Antibacterial Activity Assessment

The antibacterial activity results against S. aureus are presented in Table 3. The data indicate that while the matrix itself exhibited inert properties against microbes, the composites did demonstrate antibacterial activity. This was achieved due to the antibacterial properties of thyme indicated in the literature, where carvacrol diminishes intracellular ATP levels [11,30]. According to established standards [31], a material is considered bacteriostatic if the log reduction is above 2, which was the case for the tested composites. It should be noted that the logarithmic reduction decreased with increasing filler content. This suggests that higher thyme content, which includes more polysaccharides and other nutrients, may promote bacterial biofilm development. However, further tests should be conducted to confirm this observation.

### 3.9. Cell Viability Assessment

**Figure 13:**
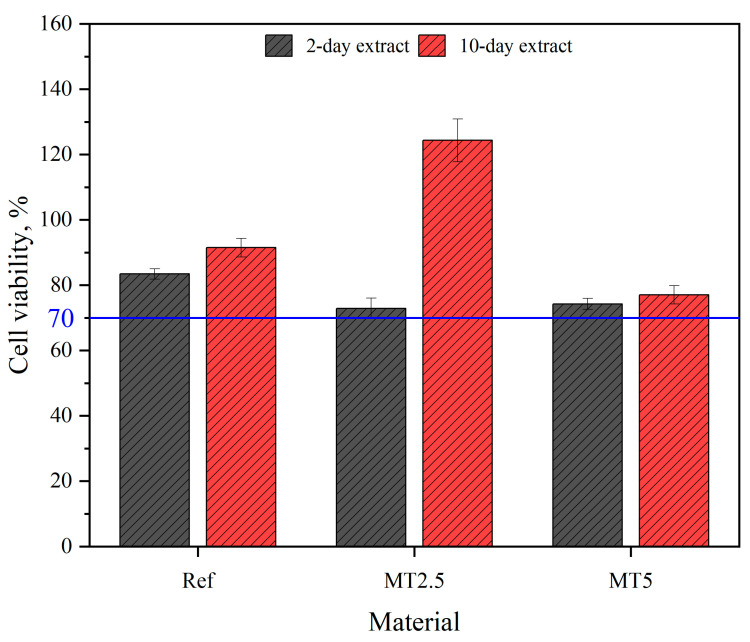
Cell viability evaluation results.

Figure 13 illustrates the viability of L-929 line cells in the 2-day and 10-day extracts of the tested materials. All materials demonstrated a viability greater than 70%, irrespective of the extraction period. Furthermore, the 10-day extracts exhibited higher cell viability than the 2-day extracts. This finding suggests that no cytotoxic components or monomers were released from the materials. Notably, the viability increased by approximately 70% for the MT2.5 10-day extract. This increase could be attributed to the presence of Thymus vulgaris, which is rich in tannins and thymine, compounds known to induce cytokine production and consequently promote cell proliferation [32]. However, beyond a specific dosage—specifically 2.5 wt.%—this effect was reversed, resulting in a significantly lower cell proliferation rate and consequently reduced cell viability.

## 4. Conclusions

Within the study’s limitations and based on the research, two biocomposites filled with 2.5 and 5 wt.% of thyme incorporated into PDMS exhibiting bacteriostatic properties were obtained. Moreover, it can be concluded that incorporating thyme affects the matrix operational properties to a varying degree, depending on the filler content.

The filler’s morphology and size enhanced integration with the matrix, contributing to higher mechanical properties. Phytochemical analysis confirmed the presence of phenolic compounds and terpenes, which influenced the composites’ bioactive properties.

Contact angle measurements showed that thyme addition retained PDMS’s hydrophobicity, which is beneficial for reducing microbial attachment. Absorption tests indicated higher rates in composites due to the filler’s hygroscopic nature. FTIR and DSC analyses revealed that thyme affected the intensity of specific peaks and crystallinity, which may impact mechanical properties.

The hardness decreased with higher thyme content due to terpene-induced polymerization inhibition, with significant differences confirmed. Tensile testing showed reduced stress at break and increased elongation with thyme addition, supporting wound dressing properties.

Antibacterial activity assessment demonstrated enhanced bacteriostatic properties in composites. Cell viability tests indicated high viability (>70%), with no cytotoxic components released. Increased viability at 2.5 wt.% thyme is attributed to cytokine-inducing compounds, though higher content reduces cell proliferation.

Future research should focus on optimizing the formulations of herb-based composites, scaling up production processes, and conducting comprehensive clinical trials to realize their full potential in wound care. By doing so, PDMS composites modified with organic fillers could become a standard in wound dressing technology, offering better healing outcomes for patients.

## 5. Patents

The findings of this research are the subject of a Polish patent application number P.448935.

## Figures and Tables

**Table 1:** Artificial plasma composition [19].

Chemical Name	Chemical Formula	Concentration (g/L)
Sodium chloride	NaCl	6.800
Potassium chloride	KCl	0.400
Calcium chloride	CaCl_2_	0.200
Magnesium sulphate	MgSO_4_	0.100
Sodium hydrogen carbonate	NaHCO_3_	2.200
Sodium hydrogen phosphate	Na_2_HPO_4_	0.126
Sodium hypophosphite	NaH_2_PO_2_	0.026

**Table 2:** Crystallinity degree results.

Material	Degree of Crystallinity (%)
Ref	40.09
MT2.5	41.98
MT5	41.23

**Table 3:** Antibacterial activity evaluation results.

Material	Log of Reduction (R)
Ref	0.06
MT2.5	2.78
MT5	2.09

## Data Availability

Not applicable.

## Supplementary Materials

The following supporting information can be downloaded at: https://www.mdpi.com/article/10.3390/ma17174224/s1. Table S1. UHPLC-QTOF-MS/MS data of compounds detected in thyme.
