# Supplementary material for: Development of Thyme-Infused Polydimethylsiloxane Composites for Enhanced Antibacterial Wound Dressings
Source: Materials (Basel). 2024 Aug 27;17(17):4224. doi: 10.3390/ma17174224 (PMC11396752; doi:10.3390/ma17174224)
Supplement: Supplementary file 1 [file materials-17-04224-s001.zip › materials-3097121-supplementary.pdf]

Table S1. UHPLC-QTOF-MS/MS data of compounds detected in thyme.

**Peak No.**	**RT (min)**	**$[M-H]^-$, *m/z***	**MS/MS fragments**	**Formula $[M-H]^-$**	**Identity**	**Reference**
1	1.02	282.0841	150.0415	$C_9H_{15}O_{10}$	unidentified	
2	2.12	359.0984	197.0457, 359.0982, 179.0350	$C_{15}H_{19}O_{10}$	syringic acid hexoside	[2]
3	2.69	315.1094	153.0558	$C_{14}H_{19}O_8$	protocatechuic acid glucoside	[2]
4	3.21	167.0353	123.0457	$C_8H_7O_4$	vanillic acid	
5	3.79	137.0236	119.0286	$C_7H_5O_3$	salicylic acid	
6	3.95	341.0883	281.0668, 221.0455, 179.0354	$C_{15}H_{17}O_9$	caffeic acid-*O*-hexoside isomer 1	[1]
7	4.33	299.0765	137.0232, 299.0764	$C_{13}H_{15}O_8$	hydroxy benzoic acid-*O*-hexoside	[2]
8	4.44	341.0885	281.0671, 221.0458, 179.0355	$C_{15}H_{17}O_9$	caffeic acid-*O*-hexoside isomer 2	[1]
9	5.07	325.0922	163.0395, 119.0501	$C_{15}H_{17}O_8$	p-coumaroyl quinic acid	[3]
10	5.2	179.0352	135.0444	$C_9H_7O_4$	caffeic acid	[3]
11	5.81	305.0699	305.0699, 225.1125	$C_{15}H_{14}O_6$	gallocatechin	[2], [4]
12	6.24	327.1499	164.0840, 327.1449, 207.1028	$C_{16}H_{23}O_7$	unidentified	
13	6.46	355.1034	193.0506	$C_{16}H_{19}O_9$	ferulic acid 4-*O*-glucoside isomer 1	[4]
14	6.64	387.1659	207.102	$C_{18}H_{27}O_9$	tuberonic acid hexoside	[3], [5]
15	6.8	377.0881	197.0453, 161.0239, 359.0766	$C_{18}H_{17}O_9$	salvianic acid C	[5]
16	6.86	385.1866	223.1332, 205.1234, 153.0911	$C_{17}H_{21}O_{10}$	sinapoyl hexoside	
17	6.92	593.1508	353.0662, 473.1087, 383.0738	$C_{27}H_{29}O_{15}$	isovitexin *C*-hexoside	[5]
18	7.55	449.1092	287.0559, 151.0031, 313.0564	$C_{21}H_{21}O_{11}$	eriodictyol hexoside isomer 2	[6]
19	8.32	463.0882	301.0349	$C_{21}H_{19}O_{12}$	quercetin *O*-hexoside	[5]
20	8.66	447.0937	285.0402	$C_{21}H_{19}O_{11}$	luteolin *O*-hexoside isomer 1	[5]
21	9.59	449.1099	287.0561, 151.0028, 313.0573	$C_{21}H_{21}O_{11}$	eriodictyol hexoside isomer 3	[6]
22	10.05	549.0896	300.0278, 463.0888	$C_{24}H_{21}O_{15}$	quercetin 3-*O*-(6”-malonyl-glucoside)	[4]
23	10.52	521.1308	323.0775, 359.0782, 197.0458	$C_{24}H_{25}O_{13}$	rosmarinic acid-3-*O*-glucoside	[7], [13]
24	10.78	717.1471	339.0513, 475.1040, 243.0299	$C_{36}H_{29}O_{16}$	salvianolic acid B	[4]
25	10.87	331.1766	287.1438	$C_{16}H_{27}O_7$	carnosic acid	[9]
26	11.29	471.1878	165.0922, 309.1350	$C_{22}H_{31}O_{11}$	unidentified	
27	11.4	359.0782	197.0459, 161.0245, 179.0354	$C_{18}H_{15}O_8$	rosmarinic acid isomer 1	[5]

28	11.59	445.0781	269.0458	$C_{21}H_{17}O_{11}$	apigenin *O*-hexsuronoside	[9], [5]
29	12.07	359.0765	197.0450, 161.0238, 179.0348	$C_{18}H_{15}O_{8}$	rosmarinic acid isomer 2	[5]
30	12.23	555.1153	161.0245, 197.0459, 359.0780, 295.0617	$C_{27}H_{23}O_{13}$	salvianolic acid K	[1]
31	12.76	371.1342	163.0759, 205.0505, 327.0870	$C_{17}H_{23}O_{9}$	syringin	[5]
32	13.98	343.0833	181.0514, 161.0247, 325.0724	$C_{18}H_{15}O_{7}$	cirsilineol	[10]
33	14.02	287.0567	151.0036	$C_{15}H_{11}O_{6}$	eriodictyol	[1], [8]
34	14.74	569.1289	161.0246, 193.0513, 339.0518, 269.0821	$C_{28}H_{25}O_{13}$	unidentified	
35	14.91	563.2155	387.1675	$C_{28}H_{35}O_{12}$	medioresinol-glucuronide	[8]
36	15.85	357.1547	149.0960, 161.0444, 113.0244	$C_{17}H_{25}O_{8}$	unidentified	
37	16.41	271.0606	151.0028	$C_{15}H_{11}O_{5}$	naringenin	[11]
38	16.49	551.1189	193.0503, 339.0505	$C_{28}H_{23}O_{12}$	clinopodic acid F	[12]
39	18.01	327.2173	171.1026, 229.1444, 211.1333	$C_{18}H_{31}O_{5}$	trihydroxyoctadecadienoic acid	[5]
40	18.18	327.2172	211.1336, 229.1441,	$C_{18}H_{31}O_{5}$	trihydroxyoctadecadienoic acid	[5]
41	18.85	553.1352	135.0445, 179.0351, 373.0630	$C_{28}H_{25}O_{12}$	unidentified	
42	19.37	329.2332	229.1443, 211.1336	$C_{18}H_{33}O_{5}$	trihydroxyoctadecenoic acid	[5]
43	19.88	287.2225	287.2225	$C_{16}H_{31}O_{4}$	dihydroxypalmitic acid	
44	21.07	424.0796	135.0442, 179.0350, 161.0239, 359.0767	$C_{44}H_{32}O_{18}$	unidentified	
45	23.43	329.1757	286.1209	$C_{20}H_{25}O_{4}$	carnosol	[4]
46	25.09	293.212	275.2015, 171.1029	$C_{18}H_{29}O_{3}$	oxooctadecadienoic acid isomer 1	[5]
47	25.17	293.2125	275.2009, 171.1024	$C_{18}H_{29}O_{3}$	oxooctadecadienoic acid isomer 2	[5]
48	25.64	559.3124	277.2163	$C_{28}H_{47}O_{11}$	fatty acid derivative	
49	25.88	295.2274	277.2168, 171.1026	$C_{18}H_{31}O_{3}$	hydroxyoctadecadienoic acid	[1]

References:

1. Luca, S. V., Zengin, G., Sinan, K. I., Skalicka-Woźniak, K., Trifan, A. (2023). Post-distillation by-products of aromatic plants from *Lamiaceae* family as rich sources of antioxidants and enzyme inhibitors. *Antioxidants*, 12(1), 210.

2. Elshibani, F. A., Mohammed, H. A., Abouzied, A. S., Abdulkarim, A. K., Khan, R. A., Almahmoud, S. A., Huwaimel B., Alamami, A. D. (2023). Phytochemical and biological activity profiles of *Thymbra linearifolia*: An exclusively native species of Libyan Green mountains. *Arabian Journal of Chemistry*, 16(6), 104775.
3. Serrano, C. A., Villena, G. K., Rodríguez, E. F., Calsino, B., Ludeña, M. A., Ccana-Ccapatinta, G. V. (2023). Phytochemical analysis for ten *Peruvian Mentheae* (*Lamiaceae*) by liquid chromatography associated with high resolution mass spectrometry. *Scientific Reports*, 13(1), 10714.
4. Ali, A., Bashmil, Y. M., Cottrell, J. J., Suleria, H. A., Dunshea, F. R. (2021). Lc-ms/ms-qtof screening and identification of phenolic compounds from Australian grown herbs and their antioxidant potential. *Antioxidants,* 10(11), 1770.
5. Sarraj, S., Szymiczek, M., Jędrejek, D., Soluch, A., Kurpanik, R. (2024). Sage-modified polydimethylsiloxane applied as antibacterial wound dressing material. *Polimery*, 69(1), 33-43.
6. Bendif, H., Peron, G., Miara, M. D., Sut, S., Dall'Acqua, S., Flamini, G., Maggi, F. (2020). Total phytochemical analysis of *Thymus munbyanus* subsp. coloratus from Algeria by HS-SPME-GC-MS, NMR and HPLC-MSn studies. *Journal of Pharmaceutical and Biomedical Analysis*, 186, 113330.
7. Borrás Linares, I., Arráez-Román, D., Herrero, M., Ibánez, E., Segura-Carretero, A., Fernández-Gutiérrez, A. (2011). Comparison of different extraction procedures for the comprehensive characterization of bioactive phenolic compounds in *Rosmarinus officinalis* by reversed-phase high-performance liquid chromatography with diode array detection coupled to electrospray time-of-flight mass spectrometry. *Journal of Chromatography A*, 1218, 7682-7690.
8. Mena, P., Cirlini, M., Tassotti, M., Herrlinger, K. A., Dall'Asta, C., Del Rio, D. (2016). Phytochemical profiling of flavonoids, phenolic acids, terpenoids, and volatile fraction of a rosemary (*Rosmarinus officinalis* L.) extract. *Molecules*, *21*(11), 1576.
9. Koutsoulas, A., Čarnecká, M., Slanina, J., Tóth, J., Slaninová, I. (2019). Characterization of phenolic compounds and antiproliferative effects of *Salvia pomifera* and *Salvia fruticosa* extracts. *Molecules*, *24*(16), 2921.
10. Grayer, R. J., Bryan, S. E., Veitch, N. C., Goldstone, F. J., Paton, A., Wollenweber, E. (1996). External flavones in sweet basil, *Ocimum basilicum*, and related taxa. *Phytochemistry*, 43(5), 1041-1047.
11. Khalil, N., Fekry, M., Bishr, M., El-Zalabani, S., Salama, O. (2018). Foliar spraying of salicylic acid induced accumulation of phenolics, increased radical scavenging activity and modified the composition of the essential oil of water stressed *Thymus vulgaris* L. *Plant Physiology and Biochemistry*, 123, 65-74.
12. Moghadam, S. E., Ebrahimi, S. N., Gafner, F., Ochola, J. B., Marubu, R. M., Lwande, W., Haller, B. F., Salehi, P., Hamburger, M. (2015). Metabolite profiling for caffeic acid oligomers in *Satureja biflora*. *Industrial Crops and Products*, 76, 892-899.
13. Ren, Q., Ding, L., Sun, S. S., Wang, H. Y., Qu, L. (2017). Chemical identification and quality evaluation of *Lycopus lucidus* Turcz by UHPLC-Q-TOF-MS and HPLC-MS/MS and hierarchical clustering analysis. *Biomedical Chromatography*, 31(5), e3867.
